# Amplification and Re-Generation of LNA-Modified Libraries

**DOI:** 10.3390/molecules171113087

**Published:** 2012-11-05

**Authors:** Holger Doessing, Lykke H. Hansen, Rakesh N. Veedu, Jesper Wengel, Birte Vester

**Affiliations:** 1 Nucleic Acid Center, Department of Biochemistry and Molecular Biology, University of Southern Denmark, 5230 Odense M, Denmark; Email: hodo@biosustain.dtu.dk (H.D.); lhh@bmb.sdu.dk (L.H.H.); 2 The Novo Nordisk Foundation Center for Biosustainability, Technical University of Denmark, Fremtidsvej 3, 2970 Hoersholm, Denmark; 3 Nucleic Acid Center, Department of Physics and Chemistry, University of Southern Denmark, 5230 Odense M, Denmark; Email: rakesh@uq.edu.au (R.N.V.); jwe@ifk.sdu.dk (J.W.); 4 School of Chemistry & Molecular Biosciences, University of Queensland, St Lucia, Brisbane, 4072 Queensland, Australia

**Keywords:** locked nucleic acid (LNA), *in vitro* selection, systematic evolution of ligands by exponential enrichment (SELEX), aptamer

## Abstract

Locked nucleic acids (LNA) confer high thermal stability and nuclease resistance to oligonucleotides. The discovery of polymerases that accept LNA triphosphates has led us to propose a scheme for the amplification and re-generation of LNA-containing oligonucleotide libraries. Such libraries could be used for *in vitro* selection of e.g., native LNA aptamers. We maintained an oligonucleotide library encoding 40 randomized positions with LNA ATP, GTP, CTP, and TTP for 7 rounds of ‘mock’ *in vitro* selection in the absence of a target and analyzed the sequence composition after rounds 1, 4 and 7. We observed a decrease in LNA-A content from 20.5% in round 1 to 6.6% in round 7. This decrease was accompanied by a substantial bias against successive LNA-As (poly-LNA adenosine tracts) and a relative over-representation of single LNA-As. Maintaining a library with LNA TTP yielded similar results. Together, these results suggest that dispersed LNA monomers are tolerated in our *in vitro* selection protocol, and that LNA-modified libraries can be sustained for up to at least seven selection rounds, albeit at reduced levels. This enables the discovery of native LNA aptamers and similar oligonucleotide structures.

## 1. Introduction

Aptamers are short DNA or RNA oligonucleotides with defined tertiary structures that enable them to act as ligands against specific target molecules. There are many applications for aptamers in biotechnology and therapeutics, which have been reviewed elsewhere [1,2]. Most aptamers are identified by *in vitro* selection (also called ‘systematic evolution of ligands by exponential enrichment’, SELEX) [3,4] in which an oligonucleotide library is incubated with the target of interest and partitioned into binders and non-binders. The binding fraction is then amplified to generate an enriched library. The best binding members are obtained by repeating this procedure under increasingly stringent conditions. 

Although aptamers can be modified to improve their stability against nucleases or confer new properties, such alterations are not straightforward and may lead to decreased affinity and/or specificity. It is therefore desirable to select aptamers from a library that already contains the desired modifications. Locked nucleic acids (LNA) are nucleotide analogues that confer thermal stability to nucleic duplexes [5,6,7,8] and nuclease resistance [8,9,10,11,12]. Some aptamers have been modified with LNA after selection [13,14,15,16,17,18,19,20], but native LNA-containing aptamers have yet to be realized. 

The synthesis of LNA ATP, LNA TTP, LNA 5-methyl-CTP, and LNA GTP [21,22,23] has opened up the field of enzymatic applications, and we [21,22,23,24,25,26] and others [27] have previously established how Phusion High Fidelity DNA polymerase and KOD DNA polymerase are both able to use an LNA-modified DNA template as well as incorporate LNA into DNA strands. Here, we demonstrate that LNA-modified libraries are suitable for *in vitro* selection experiments. 

We propose a scheme for amplification and re-generation of a fully randomized pool of LNA-containing oligomers. Using LNA ATP and LNA TTP we demonstrate how our library’s composition and overall LNA content slowly changes to include fewer and more dispersed LNA moieties over the course of several rounds of ‘mock’ *in vitro* selection. We conclude that our protocol is suitable for a limited number of rounds of *in vitro* selection to obtain, e.g., native LNA aptamers.

## 2. Results and Discussion

### 2.1. Library Amplification and Re-Generation

It has previously been demonstrated that LNA-modified templates can be read by Phusion High Fidelity DNA polymerase [25,27] and that LNA triphosphates can be used as a substrate by KOD DNA polymerase [22,23,27]. In principle, this paves the way for *in vitro* selection experiments using LNA-modified libraries. We have devised the following setup that allows us to amplify a DNA library with all adenosines replaced by LNA-A and then re-generate the single-stranded LNA-modified pool:

1. Our starting DNA library encodes 40 randomized positions. The flanking primer-binding sites do not contain adenosines.
2. The library is amplified by Phusion HF DNA polymerase under standard PCR reaction conditions to generate the corresponding double-stranded all-DNA library.
3. We then add a shorter forward primer and perform 15 rounds of primer extension using KOD XL DNA polymerase in the presence of dGTP, dCTP, dTTP, and LNA ATP. This (re-)generates the LNA-modified pool.
4. The full-length LNA-containing products are purified from a denaturing acrylamide gel. This step exploits the dissimilar lengths of the extension products and the PCR product.
5. The LNA-modified strands can then be subjected to *in vitro* selection and eventually used as PCR template for the next round.

### 2.2. LNA-A Levels Decrease over Seven Rounds

We wanted to know whether our setup was able to sustain an LNA-A modified library, or whether the foreign nature of the LNA moieties would result in loss of the LNA-containing members. We therefore performed seven rounds of mock *in vitro* selection in the absence of a specific target. By doing seven rounds we emulate a typical *in vitro* selection experiment, but any changes in the library’s composition are solely due to the intrinsic selection pressure of our setup.

By using different barcoded PCR primers for rounds 1, 4, and 7, we could pool samples from these rounds for 454 pyrosequencing. After appropriate sorting and filtering of the sequence data we obtained 19406 (round 1), 16427 (round 4) and 1529 (round 7) sequences corresponding to the 40-mer randomized region in our library. The lower number of sequences from round 7 is likely due to a dilution error.

**Figure 1:**
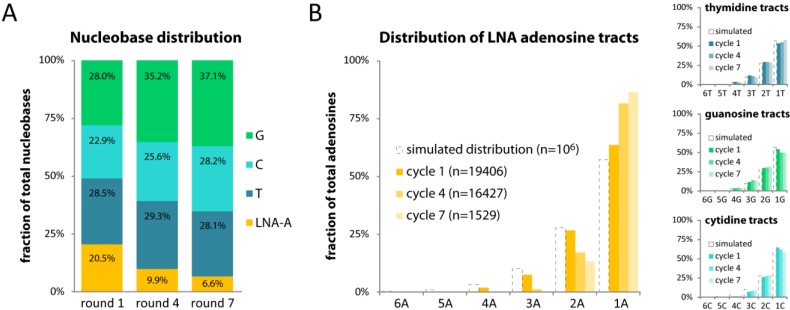
(**A**) Nucleobase distribution in the library’s 40-mer randomized region after one, four and seven rounds of mock *in vitro* selection; (**B**) The distribution of homopolymer tracts of each nucleobase in the randomized 40-mer region after one, four, and seven cycles of mock *in vitro* selection. Dotted bars indicate the reference distribution of tract lengths as obtained from simulating 1 million random 40-mer sequences.

According to our 454 sequencing data the overall LNA-A content dropped 2-fold from 20.5% in round 1 to 9.9% in round 4 and further 1.5-fold to 6.6% in round 7 (Figure 1A). Concomitantly, thymidine levels were near constant (at 28%), cytidine levels somewhat elevated (22.9% to 28.2%), and guanosine levels increased from 28.0% to 37.1%.

### 2.3. Single LNA-As are Favored over Successive LNA-As

We speculated if successive LNA moieties pose a particular challenge in our setup and looked at the frequency of various lengths of poly-adenosine tracts. The dotted bars in Figure 1B indicate the distribution found in 1 million *in silico*-generated 40-mer sequences and acts as a reference. The distribution of adenosine tracts after round 1 corresponded well with the simulated distribution (Figure 1B, left panel). However, after round 4 we found a significant decrease of at least 35% in the number of poly-adenosine tracts (AA (2A), AAA (3A), AAAA (4A) *etc*.), whereas single adenosines (1A) increased in frequency by 28%. This tendency became even more pronounced after round 7. Notably, we only saw this trend with LNA-A, as the relative frequencies of thymidine, cytidine and guanosine tracts were practically unaffected between rounds 1, 4, and 7 (Figure 1B, right panels). This suggests that single LNA moieties are favoured over successive LNAs.

### 2.4. LNA-T also Skews Library Composition

**Figure 2:**
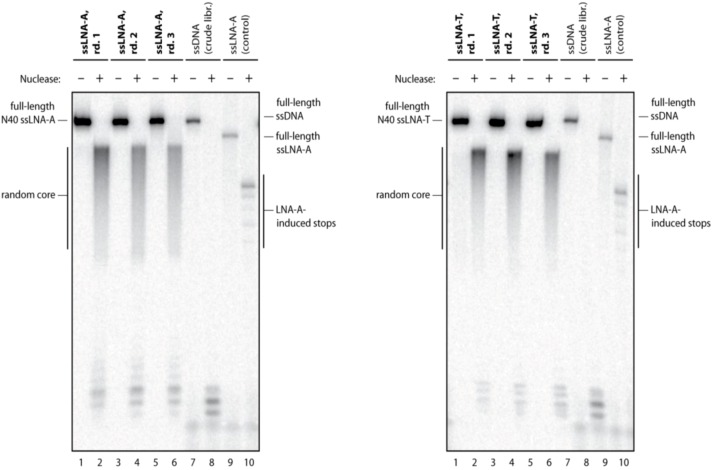
(**Left**) Exonuclease digestion of the LNA-A containing library after 1, 2, and 3 rounds of mock *in vitro* selection (lanes 1–6). In the presence of exonuclease (+), the 3' end is readily degraded, while the LNA-containing random core is partially protected. The crude all-DNA library is readily degraded (lanes 7,8). A DNA oligomer with LNA-A at defined positions results in a step-wise degradation pattern (lane 9, 10). (**Right**) As in the left panel, but with exonuclease digestion of the LNA-T containing library after 1, 2, and 3 rounds of mock *in vitro* selection (lanes 1–6).

We also asked whether the changes in LNA content and context were specific to LNA-A. To this end, we compared libraries maintained with LNA ATP and LNA TTP. We used an oligo-capture strategy for purification of these LNA-modified libraries; details can be found in the Experimental section. Digestion of the libraries suggested slightly increasing exonuclease susceptibility and thus decreasing LNA content over the first three rounds, regardless of the type of LNA nucleotide (Figure 2). After three rounds of mock *in vitro* selection we cloned and Sanger sequenced between 27 and 43 clones from each library.

**Figure 3:**
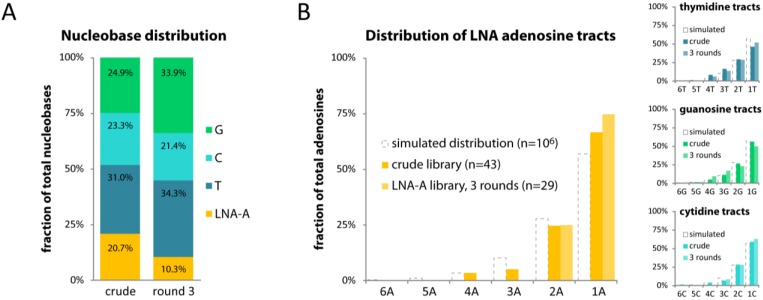
(**A**) Nucleobase distribution in the library’s 40-mer randomized region before and after being maintained with LNA ATP for three rounds of mock *in vitro* selection; (**B**) The distribution of homopolymer tracts of each nucleobase in the randomized 40-mer region in the crude DNA library or after three cycles of mock *in vitro* selection using LNA ATP. Dotted bars indicate the reference distribution of tract lengths as obtained from simulating 1 million random 40-mer sequences.

The library maintained with LNA ATP showed a 2-fold drop in total adenosines (20.7% to 10.3%; Figure 3A) in the randomized 40-mer region, and single adenosines increased in numbers, while tracts of 3 or more adenosines became absent (Figure 3B). This behaviour was very similar to that observed with LNA ATP in our 454 sequencing data (Figure 1), although the number of members evaluated by Sanger sequencing was much smaller.

**Figure 4:**
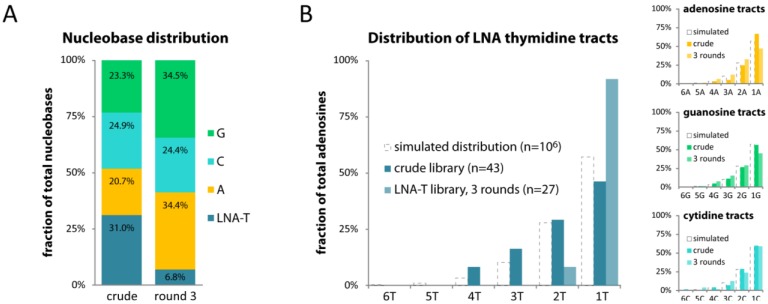
(**A**, **B**) As in Figure 3, except that the library was maintained with LNA TTP, using its complementary sequence as initial template.

The library maintained with LNA TTP showed a remarkable 4.5-fold drop in total thymidines (31.0% to 6.8%; Figure 4A). The ratio of single thymidines increased 2-fold, while tracts of two or more thymidines were greatly reduced or entirely absent. Conversely, the distributions of adenosine, guanosine and cytidine tracts corresponded well with their simulated distributions (Figure 4B). Overall, our data suggest a similar or stronger skewing of LNA content in a library maintained with LNA TTP compared to that maintained with LNA ATP.

## 3. Experimental

### 3.1. Library and Primers

Library and primers: 5'-GCTCTGTCGTCGTGGTCGGTCC-(N_40_)-GGCCTTTTGTGTGTCGTTT, 5'-GCTCTGTCGTCGTGGTCGGTCC, 5'-AAACGACACACAAAAGGCC. Barcoding forward primers for 454 sequencing were 5'-CGTATCGCCTCCCTCGCGCCATCAGCTCTGTCGTCGTGG­TCGGTCC (rd. 1–3), 5′-CGTATCGCCTCCCTCGCGCCATCAGCTGTCGTCGTGGTCGGTCC (rd. 4–6), and 5'-CGTATCGCCTCCCTCGCGCCATCAGCGCTCTGTCGTCGTGGTCGGTCC (rd. 7). Synthesis of LNA-A containing libraries was in the forward direction, while synthesis of LNA-T containing libraries was in the reverse direction, using the complementary strand as template.

### 3.2. Polymerase Chain Reaction (PCR)

5 nM template and 0.5 µM of each primer was combined in 1× Phusion HF buffer with 200 µM of each deoxyribonucleotide triphosphate and 0.02 units/µL Phusion DNA polymerase (Finnzymes, Espoo, Finland). Typical PCR conditions were: 98 °C/5 min, 24 cycles (98 °C/5 s, 53 °C/10 s, 72 °C/5 min), 4 °C/hold. Sanger sequencing experiments used 0.5 nM template and 0.04 units/µL Phusion DNA polymerase over 20 cycles.

### 3.3. Primer Extension with LNA

Reaction conditions were: 1 × KOD XL buffer, 3 mM MgSO_4_, 0.2 mg/mL BSA, 0.2 units/µL KOD XL DNA polymerase (Novagen), 2.5 µM primer, 2.5 ng/µL dsDNA, and 0.25 mM of each nucleotide triphosphate as required; LNA triphosphates were synthesized at the Nucleic Acid Center. Thermocycling was: 95 °C/1 min, 15 cycles (95 °C/15 s, 60 °C/35 s, 72 °C/5 min), 4 °C/hold. Reactions were quenched with excess EDTA and purified from denaturing 6% polyacrylamide gels by crush-and-soak.

### 3.4. Sequencing and Analysis

Aliquots of LNA-A-modified libraries after 1, 4 and 7 selection rounds were barcoded by PCR and pooled. 454 GS FLX-sequencing was by GATC Biotech (Solna, Sweden). Filtering parameters were adjusted for short amplicons by GATC Biotech. The amplicons were sorted by barcode and the randomized regions were extracted and analyzed using custom Python scripts. Incomplete regions or members with low 454 quality scores were removed. Tract distribution was simulated by generating and analyzing 1 million random 40-mers.

### 3.5. Alternate strand Isolation

We used an oligo capture approach for libraries for Sanger sequencing: PCR by Phusion HF DNA polymerase was with a 5'-phosphorylated primer to enable the removal of the forward strand by lambda exonuclease. The resulting single-stranded template was then used with KOD DNA polymerase and the appropriate triphosphates. Only full-length products were recovered by hybridizing to a biotinylated capture probe specific for the 3' end of the product and subsequent capture on Streptavidin-coated paramagnetic beads. After careful washing the pure product strands were heat-eluted.

### 3.6. Lambda Exonuclease Digestion

Double-stranded DNA was prepared by PCR with a 5'-phosphorylated primer and a 5' fluorescein-labeled primer. Digestion with lambda exonuclease (New England Biolabs, Ipswich, MA, USA) was with 6.7 units/µg double-stranded DNA (50 ng/µL final DNA concentration) at 37 °C for 25 min. The reaction was stopped by addition of 1 volume 50 mM EDTA or by heating to 75 °C for 15 min. Full digestion was verified by agarose gel electrophoresis and ethidium bromide staining. Fluorescence scanning was on a Typhoon phosphoimager (GE Healthcare, Piscataway, NJ, USA).

### 3.7. Oligonucleotide Capture on Beads

Primer extension products were slow-annealed over 30 min. or more with at least 2-fold molar excess of dual biotinylated oligomer complementary to the products’ 3' end (5'-[biotin]-AAACGACACACAAAAGGCC). The solution was adjusted to 1 × B&W (according to manufacturer’s instructions) and combined with Dynabeads M-280 Streptavidin (Invitrogen, Life Technologies Europe BV, Naerum, Denmark). The number of beads corresponded to an excess of biotin binding sites. After 15 min with gentle agitation the beads were washed first in 1 × B&W, then in 1 × SSC, before heat-elution of the non-biotinylated species into 40 µL 1 × SSC (75 °C, 5 min).

### 3.8. Digestion Assay

The DNA library, the LNA-containing libraries, and a synthetic LNA-A containing oligomer (5'-GGTCTGGTCCACACCCAGCCGCCaCCCaGGGaCGCaGCCaGGCaCGGCGGGCCTATAGT­GAGTCGTATTA; lowercase is LNA-A) were 5'-radiolabelled with T4 polynucleotide kinase (Fermentas) and gel purified. The oligos were incubated at 72 °C in digestion buffer (1 × KOD buffer #2, 3 mM MgSO_4_, 0.2 mg/mL BSA) with or without 0.02 units/µL KOD DNA polymerase (Novagen, San Francisco, CA, USA). Reactions were quenched in 1 vol. ice-cold 95% formamide/50 mM EDTA and resolved on 13% denaturing polyacrylamide gels. Autoradiography was on a Typhoon phosphoimager (GE Healthcare).

### 3.9. Sanger Sequencing

PCR products were blunt-end cloned into SmaI-digested pUC18 or pUC19 (this offered multiple inserts per plasmid) and transformed into *E. coli* TOP10 (Invitrogen). Plasmids from re-streaked colonies were purified (Miniprep, Qiagen, Valencia, CA, USA) and sequenced by Eurofins MWG Operon (Ebersberg, Germany). Sequence analysis was with custom Python scripts.

## 4. Conclusions

This is the first report of how the capabilities of Phusion High Fidelity DNA polymerase and KOD DNA polymerase may be used in concert to amplify and re-generate LNA-containing libraries. We find that although LNA moieties present a challenge, LNA-containing libraries can be maintained over several rounds. Specifically, we see a 3-fold decrease in LNA-A content (20.5% to 6.6%, Figure 1A) over seven rounds of mock *in vitro* selection. Others have reported similar [28] or conflicting [29] sequence drifts with RNA libraries subjected to mock *in vitro* selection. However, we believe the trend observed in our data to be due to the ‘locked’ nature of the LNA nucleotide employed, as we found similar changes in thymidine levels when our library was maintained with LNA TTP. We also observe a bias against members encoding successive LNA moieties. We have previously observed difficulties in incorporation of successive LNA nucleotides [21,30], but the rationale is not clear or consistent regarding the conditions, polymerases, and nature of the LNA nucleotide.

Historically, *in vitro* selection experiments have been performed with six or more rounds. Hence, a traditional selection using our setup is feasible if the current limitations are addressed. Emulsion PCR has been shown to prevent the spurious product formation that often results after excessive amplification of DNA libraries [31,32,33]. This may potentially allow additional rounds of LNA incorporation to be performed and aid in the retention of library members with high LNA content. Conversely, more recent selection techniques based on e.g., capillary electrophoresis [34,35,36,37,38], microfluidic separation [39,40,41,42], and/or next-generation sequencing [43,44,45,46] generally require only 1–4 selection rounds and therefore seem ideally suited for selection with LNA triphosphates.

In summary, we have demonstrated that our setup allows for several rounds of amplification and re-generation of an LNA-modified library while retaining a significant number of single LNA moieties. This paves the way for *in vitro* selection with LNA-modified libraries to obtain e.g., native LNA aptamers. Our approach seems especially suited for modern selection techniques that require fewer selection rounds.
